# Developing model-based algorithms to identify screening colonoscopies using administrative health databases

**DOI:** 10.1186/1472-6947-13-45

**Published:** 2013-04-10

**Authors:** Maida J Sewitch, Mengzhu Jiang, Lawrence Joseph, Robert J Hilsden, Alain Bitton

**Affiliations:** 1 Department of Medicine, McGill University, Montreal, Quebec, Canada; 2 Division of Clinical Epidemiology, Research Institute of the McGill University Health Centre, Montreal, Quebec, Canada; 3 Division of Gastroenterology, McGill University Health Centre, Montreal, Quebec, Canada; 4 Department of Epidemiology, Biostatistics and Occupational Health, McGill University, Montreal, Quebec, Canada; 5 Departments of Medicine/Community Health Sciences, University of Calgary, Calgary, Alberta, Canada

## Abstract

**Background:**

Algorithms to identify screening colonoscopies in administrative databases would be useful for monitoring colorectal cancer (CRC) screening uptake, tracking health resource utilization, and quality assurance. Previously developed algorithms based on expert opinion were insufficiently accurate. The purpose of this study was to develop and evaluate the accuracy of model-based algorithms to identify screening colonoscopies in health administrative databases.

**Methods:**

Patients aged 50-75 were recruited from endoscopy units in Montreal, Quebec, and Calgary, Alberta. Physician billing records and hospitalization data were obtained for each patient from the provincial administrative health databases. Indication for colonoscopy was derived using Bayesian latent class analysis informed by endoscopist and patient questionnaire responses. Two modeling methods were used to fit the data, multivariate logistic regression and recursive partitioning. The accuracies of these models were assessed.

**Results:**

689 patients from Montreal and 541 from Calgary participated (January to March 2007). The latent class model identified 554 screening exams. Multivariate logistic regression predictions yielded an area under the curve of 0.786. Recursive partitioning using the latent outcome had sensitivity and specificity of 84.5% (95% CI: 81.5-87.5) and 63.3% (95% CI: 59.7-67.0), respectively.

**Conclusions:**

Model-based algorithms using administrative data failed to identify screening colonoscopies with sufficient accuracy. Nevertheless, the approach of constructing a latent reference standard against which model-based algorithms were evaluated may be useful for validating administrative data in other contexts where there lacks a gold standard.

## Background

Administrative records are frequently used in health research. While some diagnoses and procedures are recorded with reasonable accuracy [1,2], others are prone to misclassification [3,4]. Indications for medical procedures are particularly challenging to derive from administrative health data because of the lack of indication codes, and, therefore, require automated data algorithms [5-7]. Studies validating administrative data algorithms have typically used medical chart review as the gold standard [7-13]. However, information in medical charts may be inaccurate for reasons including the variable quality of record keeping and record extraction. Moreover, with Canadian average wait-times from referral to performance of any gastrointestinal procedure of 155 days and to screening colonoscopy of 201 days [14], the medical chart may not reflect symptoms at the time the colonoscopy is performed. In this study, we undertook the challenge of evaluating model-based algorithms in the absence of a gold standard measure for the indication of colonoscopy.

Colorectal cancer (CRC) screening is recommended worldwide for asymptomatic persons aged 50 to 75 [15-18]. Many industrialized countries have committed to or already implemented population-based CRC screening programs using modalities such as fecal occult blood test, fecal immunochemical test, flexible sigmoidoscopy, and colonoscopy [19]. Colonoscopy is the only exam that enables visualization and removal of precancerous and cancerous lesions throughout the entire colon, and recent guidelines promote its role as a first-line screening modality [15,16]. Colonoscopy utilization has increased dramatically owing to its use in CRC screening [20,21]. In addition to its use in screening, colonoscopy is also performed for surveillance for bowel diseases, diagnostics for large bowel symptoms, and follow-up for positive results by other CRC screening modalities. A screening colonoscopy is defined as one performed in asymptomatic individuals for the early detection of CRC or the detection and removal of precancerous lesions [22].

Population screening initiatives are accompanied by increasing interest in undertaking large-scale colonoscopic screening studies using existing administrative health databases. However, most such databases either do not have a screening colonoscopy procedure code or the code is underused since the primary purpose is remuneration [21,23]. Methods to distinguish screening and non-screening colonoscopies would enable monitoring of CRC screening uptake, tracking of health resource utilization, and estimation of cost-effectiveness; they may also be used for quality assessment as a key quality indicator is the adenoma detection rate in screening colonoscopies [22,24,25]. Automated data screening colonoscopy algorithms developed in previous studies had sensitivities ranging between 29% and 84% and specificities ranging between 58% and 93%; none of the algorithms had both high sensitivity and specificity [7,12,13], which led to the conclusion that administrative data cannot reliably be used to distinguish between colonoscopy indications [7,26]. However, these prior studies relied on expert opinion regarding which diagnostic and procedural codes to use and in what order. In contrast, model-based algorithms use the data to help determine which variables to include and their weights, and thus have the potential to be more accurate.

A validated database algorithm would be convenient and efficient for researchers and administrators. The objectives of this study were to develop model-based algorithms to identify screening colonoscopies in health administrative databases, to evaluate their accuracy against a latent reference standard, and to compare their accuracies to that from a previously developed algorithm based on expert opinion.

## Methods

### Study design

A retrospective cohort study was conducted of endoscopists and a convenience sample of their patients about to undergo colonoscopy between January and March 2007 in two Canadian cities (Montreal, Quebec and Calgary, Alberta). Participating institutions were those where colonoscopy was performed and billed to the provincial health insurance plans (Montreal: McGill University Health Centre, Sir Mortimer B. Davis Jewish General Hospital, St. Mary’s Hospital Centre, Centre hospitalier de l’Université de Montréal, Fleury Hospital, Maisonneuve-Rosemont Hospital; Calgary: Foothills Medical Centre, Peter Lougheed Centre). At the time of the study, the provincial CRC screening program in Alberta relied on family physicians to offer fecal occult blood tests to patients aged 50 to 75 but no such program existed in Quebec, where ‘screening’ occurred opportunistically at the individual physician’s discretion. In both provinces, patients and/or family physicians could opt for colonoscopy as the initial screening exam.

### Data collection

The research assistant assessed endoscopists and patients for eligibility. Eligible endoscopists received remuneration for colonoscopy from the provincial health insurance board. Immediately after each colonoscopy, the endoscopist completed a questionnaire on the colonoscopy indication; the screening indication was defined as ‘performed in asymptomatic people at average-risk for developing colorectal cancer, or in people with a family history of colorectal cancer’. It is unknown whether the endoscopist based the indication on the colonoscopy referral, communication with the patient, or something else. Eligible patients were aged 50 to 75 years; those without provincial health insurance plan coverage in the prior year or unable to give consent were excluded. The research assistant approached patients prior to colonoscopy, explained the study, obtained consent, and administered the patient questionnaire. Patient perceived indication was defined in two ways: 1) non-screening if patient reported that the reason for colonoscopy was to follow-up for a previous test or problem, and is screening otherwise; and 2) non-screening if patient reported specific lower abdominal symptoms and personal history of gastrointestinal (GI) condition, and is screening otherwise. These patient indications, and their agreement with endoscopist indication, have been described in detail elsewhere [27].

We obtained provincial administrative health data on participating patients for the five years prior to the index colonoscopy as follows: Physician billing records from the Régie de l’assurance maladie du Québec (RAMQ) and Alberta Health and Wellness provided data on patient age and sex, all medical acts (RAMQ billing codes in Quebec, and Canadian Classification of Diagnostic, Therapeutic, and Surgical Procedures (CCP) codes in Alberta). The Maintenance et Exploitation des Données pour l’Étude de la Clientèle Hospitalière (MED-Echo) and the Canadian Institute for Health Information (CIHI) provided information on hospitalizations (The International Classification of Diseases ICD-9 and ICD-10 codes) and surgeries (ICD-9, CCP, and Canadian Classification of Health Interventions (CCI) codes). Data were linked using unique patient health insurance numbers. Prior to study inception, ethics approval was obtained from the Institutional Review Board in the Faculty of Medicine at McGill University and the local research ethics boards.

### Statistical analyses

Since there is no gold standard method for identifying screening colonoscopies, a Bayesian latent class model for diagnostic testing was used to provide the probability that any given colonoscopy was for screening purposes [28], based on endoscopist indication and the two patient indications. Flat or non-informative prior distributions were used for the two patient indications, which were assumed to be conditionally independent. To examine the robustness of this assumption, a model including a dependence between these two indications was also fitted to the same data [29]. For the endoscopist screening indication, a beta(10.67, 1.06) density, 97% of which covers the range from 70% to 100%, was used for both sensitivity and specificity. A beta(6, 7.6) density, 95% of which covers the range from 20-70% was used for the prevalence of screening. These priors were based on expert opinion, and covered the ranges of all plausible values with relatively flat density. Latent class modeling was carried out using WinBUGS software (MRC Biostatistics Unit, Cambridge).

The predicted probabilities for screening from the latent class model, based on posterior medians, were dichotomized into screening and non-screening using a cut-off of 50% probability. The dichotomized latent class indication was then used as the outcome variable for multivariate logistic regression and recursive partitioning. For comparison purposes, we also fitted models using endoscopist indication alone as the outcome. Predictor variables entered into the models were: age, sex, procedure codes for previous procedures (colonoscopy, polypectomy, sigmoidoscopy, and double contrast barium enema (DCBE)) in the past 4 years, diagnostic codes for risk factors (inflammatory bowel disease (IBD), colorectal polyp, and CRC) in the past 5 years, diagnostic codes for symptoms (rectal bleeding, anemia, diarrhea, vomiting, and weight loss) in the past year, diagnostic codes for hospitalization for large bowel diseases in the past 5 years, and procedure codes for large bowel surgeries in the past 5 years (Additional file 1). The variables selected were based on practice guidelines [15,16,18], published studies [7,12,13], and expert opinion.

The Bayesian information criterion (BIC) was used to select the multivariate logistic regression model that best predicts the screening indication [30]. Model discrimination was assessed by the area under the curve (AUC) of the receiver operator characteristic curve [31]. The accuracy of classification trees generated by the recursive partitioning model was assessed against the latent class predictions and endoscopist indication; sensitivities, specificities, positive and negative predicative values (PPV, NPV) were computed. Multivariate and recursive modeling were performed using R [32].

We also applied an algorithm based on expert-opinion previously developed by El-Serag et al., which defines screening colonoscopies as the absence of ICD-9 codes for 28 symptoms or conditions and no colonoscopy in the past 4 years [12]. Sensitivities, specificities, PPVs, and NPVs were estimated by comparing the algorithm classification to latent class predictions and to endoscopist indication.

## Results

### Participant characteristics

A total of 1,411 patients were approached, of which 1,230 (87.2%) were eligible and agreed to participate, 689 (56.0%) from Montreal and 541 (44.0%) from Calgary. In Montreal and Calgary, 52 and 0 eligible patients approached refused study participation, respectively. The average age was 60 and 48.5% of participants were male (Table 1). Endoscopists reported screening as the reason for colonoscopy in 46.8% of colonoscopies, whereas patient indications 1 (patient perceived reason) and 2 (based on patient reported symptoms and GI history) were screening in 51.0% and 38.9% of the colonoscopies, respectively. The frequency of occurrence of diagnostic and procedure codes of interest in patient administrative health records are presented in Table 2.

**Table 1:** Patient characteristics (N = 1,230)

**Patient characteristic**	**N**	**%**
Age (mean, sd)	60.1 (6.9)	
Male sex	597	48.5
Patient reported symptoms^a^	573	46.6
Patient reported gastrointestinal conditions^b^	350	28.5
Patient reported positive FOBT^c^ in the past 12 months	71	5.8
Endoscopist indication = screening	576	46.8
Patient indication 1^d^ = screening	627	51.0
Patient indication 2^e^ = screening	478	38.9

^a^ Symptoms include rectal bleeding, lower abdominal pain, unintentional weight loss, and change in bowel habits in the past 6 months, as well as anemia in the past 12 months.

^b^ Gastrointestinal conditions include colorectal cancer, colorectal polyps, inflammatory bowel disease, and previous bowel surgery.

^c^ Fecal occult blood test.

^d^ Patient indication 1 is non-screening when patient perceived reason for the colonoscopy was to follow up on a problem or a previous test, and is screening otherwise.

^e^ Patient indication 2 is non-screening when patient reports specific symptoms, personal history of gastrointestinal conditions, or recent positive FOBT, and is screening otherwise.

**Table 2:** Frequency of occurrence of diagnostic and procedure codes in provincial administrative databases (N = 1,230)

**Diagnostic or procedure codes**	**N**^**a**^	**%**
**Procedures in the past 4 years**		
Colonoscopy	267	21.7
Sigmoidoscopy	76	6.2
Polypectomy	114	9.3
Double contrast barium enema	65	5.3
**Symptoms in the past year**		
Rectal bleeding	95	7.7
Diarrhea	50	4.1
Vomiting	7	0.6
Weight loss	8	0.7
Anemia	86	7
**Gastrointestinal conditions in the past 5 years**		
Colorectal cancer	65	5.3
Colorectal polyps	205	16.7
Inflammatory bowel disease	48	3.9
**Hospitalizations in the past 5 years**		
Large bowel diseases	32	2.6
Large bowel surgery	26	2.1

^a^ Number of patients whose health administrative records contain the relevant diagnostic or procedure code(s). Specific codes are listed in Additional file 1.

### Model-based algorithms

The latent class model predicted 554 (45.0%) screening exams. The Kappa statistic for its agreement with endoscopist indication was 0.794 (95% CI: 0.760-0.828). Allowing conditional dependence between the two patient indications yielded virtually identical results (data not shown).

Using the latent class indication as the outcome, the multivariate logistic regression model that included age, sex, and all administrative data variables yielded an AUC of 0.786 (95% CI: 0.760-0.812) when the logistic model predictions were compared to the latent class indication. The best model selected by BIC had an AUC of 0.754 (95% CI: 0.726-0.782) and included 8 administrative data variables (Table 3). In comparison, multivariate logistic regression using the endoscopist indication alone as the outcome provided an AUC of 0.791 (95% CI: 0.765-0.816) for the full model and 0.761 (95% CI: 0.734-0.788) for the best model selected by BIC. The same 8 variables were selected by the BIC, with similar odds ratio estimates (Table 3).

**Table 3:** **Odds ratio estimates for the multivariate logistic regression models selected by BIC**^**a**^

**Variable**	**Latent class indication**	**Endoscopist indication**
	**OR**^**b **^**(95% CI)**	**OR (95% CI)**
Colonoscopy in the past 4 years	0.16 (0.10-0.23)	0.18 (0.12-0.25)
Sigmoidoscopy in the past 4 years	0.29 (0.17-0.45)	0.28 (0.16-0.43)
Polypectomy in the past 4 years	0.24 (0.12-0.42)	0.13 (0.05-0.28)
DCBE^c^ in the past 4 years	0.19 (0.11-0.31)	0.19 (0.11-0.31)
Rectal bleeding in the past year	0.15 (0.09-0.25)	0.12 (0.07-0.20)
Diarrhea in the past year	0.20 (0.11-0.34)	0.14 (0.07-0.27)
Anemia in the past year	0.15 (0.09-0.24)	0.19 (0.12-0.30)
IBD^d^ in the past 5 years	0.06 (0.02-0.23)	0.09 (0.03-0.24)

^a^ Bayesian information criterion.

^b^ Odds ratio adjusted for all 7 other variables in the model.

^c^ Double contrast barium enema.

^d^ Inflammatory bowel disease.

**Figure 1:**
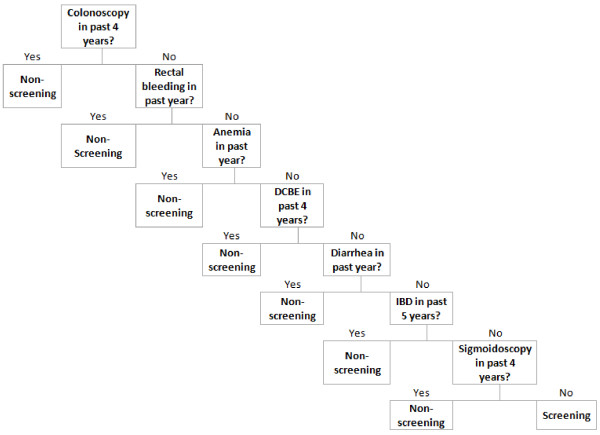
**Classification tree for colonoscopy indication generated by recursive partitioning model using latent class predictions as the outcome.** Colonoscopy exams were classified as screening or non-screening based on the presence or absence of diagramed diagnostic or procedure codes in patient administrative health records. DCBE: double contrast barium enema. IBD: inflammatory bowel disease.

Recursive partitioning using the latent class indication as the outcome used 7 variables, yielding the classification tree in Figure 1. The sensitivity and specificity, when comparing the classification tree to the latent class indication as the reference standard, were 84.5% and 63.3% respectively (Table 4). Recursive partitioning using the endoscopist indication as the outcome yielded a similar classification tree but used only 6 variables in the following order:: colonoscopy in the past 4 years, rectal bleeding in the past year, DCBE in the past 4 years, diarrhea in the past year, anemia in the past year, and IBD in the past 5 years. Sensitivity was 85.1% and specificity was 62.2% (Table 4).

**Table 4:** Accuracy measures for recursive partitioning and expert opinion algorithms

**Algorithm**	**Reference standard**	**Sensitivity%**	**Specificity%**	**PPV **^**a**^**%**	**NPV **^**b**^**%**
		**(95% CI)**	**(95% CI)**	**(95% CI)**	**(95% CI)**
Recursive partitioning with latent class outcome	Latent class indication	84.5	63.3	65.4	83.3
		(81.5-87.5)	(59.7-67.0)	(61.9-68.9)	(80.0-86.5)
Recursive partitioning with endoscopist outcome	Endoscopist indication	85.1	62.2	66.5	82.6
		(82.2-88.0)	(58.5-66.0)	(63.1-70.0)	(79.2-85.9)
El-Serag	Latent class indication	49.2	82.0	69.1	66.4
		(45.1-53.4)	(79.1-84.9)	(64.6-73.7)	(63.3-69.6)
El-Serag	Endoscopist indication	49.3	83.0	71.9	65.0
		(45.2-53.4)	(80.2-85.9)	(67.5-76.3)	(61.8-68.3)

^a^ Positive predicative value.

^b^ Negative predictive value.

### Expert opinion algorithm

The algorithm developed by El-Serag et al. was applied to our data. The algorithm identified 395 (32.1%) colonoscopies as screening. The sensitivity and specificity were 49.3% and 82.0%, respectively, compared to the latent class indication, and 49.3% and 83.0% compared to the endoscopist indication (Table 4).

## Discussion

We evaluated model-based algorithms in the absence of a gold standard measure of the outcome for determining the colonoscopy indication (screening vs. non-screening). We tackled this problem by constructing a latent reference standard and then using it to develop and evaluate logistic regression and recursive partitioning models of administrative data variables. Both modeling approaches have been used in previous studies to identify the indications for of medical procedures [6,10]. The latent class predictions were quite accurate when the various tests all agreed on the indication, i.e. when all tests together indicated either positive or negative for screening. However, when one or more tests disagreed with the others, there was higher variability and less certainty about the inputs. Overall, the stability of the logistic regression model was very good, as evidenced by the robustness of our analyses (using a second latent class model that gave very similar predictions).

From multivariate logistic models, the AUC was 0.786 and 0.791 for the full models fitted with latent and endoscopist indications as the outcomes, respectively. The greater than 20% chance of mistakenly ranking a non-screening exam as more likely to be screening than a screening exam is not sufficiently accurate for most research purposes. The recursive models also did not achieve sufficient accuracy, despite their propensity to overfit data. Compared to the expert opinion algorithm, the recursive models had higher sensitivity but lower specificity; this occurred largely because the El-Serag algorithm defined screening as the absence of 28 diagnosis and colonoscopy procedure in the past 4 years, whereas recursive partitioning chose only the most discriminating variables. However, direct comparisons between the model-based and the El-Serag algorithms should be done with caution, as the models have the advantage of being validated on the same data upon which they were constructed.

Since we had two datasets available, the prudent approach would have been to construct the models using one dataset and validate them in another. However, our intention was to show that even under optimal circumstances – using all the data to generate the models and evaluating with the same dataset – model accuracies were less than satisfactory. Not pruning the classification tree, which tends to overfit the data in the recursive model [33,34], also did not result in more accurate predictions.

Both the latent class and the endoscopist indications produced very similar results in most cases, since there was relatively high agreement between them. The latent class predictions were likely driven more by the endoscopist indication than patient indications, given the informative prior distribution used for the endoscopist indication, while uninformative priors were used for patient indications. The assumption that physicians know the true indication at least 70% of the time seems conservative.

The poor performance of algorithms, whether model-based or expert-opinion based, may be due to imperfect accuracies of administrative codes for the predictor variables we used [4] or to the variability in physician clinical and billing practices [35]. The polypectomy procedure code in Quebec, for example, underestimates the number of polypectomies by 15% [1]. Since the primary purpose of health administrative data collection is remuneration, diagnostic codes may be poorly recorded [35], leading to misclassification. Model selection in the multivariate logistic regression analysis retained all 4 procedure variables (colonoscopy, sigmoidoscopy, polypectomy, DCBE in the past 4 years) as important predictors of indication, while it retained only 4 of 8 diagnostic variables from physician billing data. Procedure codes may have been better predictors than some diagnostic codes because they were recorded with greater accuracy due to the need for remuneration [35]. CRC diagnosis and colorectal polyps were not identified as useful predictors by either logistic regression model selection procedures or recursive partitioning, possibly due to overlapping information with other variables or poor accuracy. Hospitalizations for large bowel disease and large bowel surgeries were also not selected as important predictors, possibly due to the small numbers of patients whose records contained these codes.

Algorithms are typically needed to accurately identify cases of IBD because of problems with misclassification in administrative data [9,36]. We did not use such an algorithm for IBD as the purpose was not to correctly identify IBD but to evaluate the utility of administrative codes (including those for IBD) in predicting colonoscopy indication. Despite the relatively low frequency of occurrence of IBD codes, the variable emerged as an important predictor in all models.

## Conclusions

In conclusion, model-based screening colonoscopy algorithms for administrative databases were insufficiently accurate to be used for most research purposes. However the novel approach that we have employed, constructing a latent reference standard against which model-based algorithms were evaluated, may be useful for validating administrative data in other contexts where gold standards are unavailable.

## Competing interests

There are no financial or other competing interests.

## Authors’ contributions

MJS conceived of the study, participated in the study design and coordination, and helped draft the manuscript. MJ participated in the statistical analysis and drafting of the manuscript. LJ participated in the statistical analysis and in the drafting of the manuscript. RJH participated in the study design and with acquisition of the data. AB participated in the study design and with acquisition of the data. All authors contributed to the interpretation of the findings, and read and approved the final manuscript.

## Pre-publication history

The pre-publication history for this paper can be accessed here:

http://www.biomedcentral.com/1472-6947/13/45/prepub

## Supplementary Material

Additional file 1 Diagnostic and procedure codes in health administrative databases. Click here for file
