# Supplementary material for: Developing model-based algorithms to identify screening colonoscopies using administrative health databases
Source: BMC Med Inform Decis Mak. 2013 Apr 10;13:45. doi: 10.1186/1472-6947-13-45 (PMC3637812; doi:10.1186/1472-6947-13-45)
Supplement: Additional file 1 — Diagnostic and procedure codes in health administrative databases. [file 1472-6947-13-45-S1.doc]

Diagnostic and procedure codes in health administrative databases

| **Procedures in the past 4 years** | **RAMQ** ^a^ **billing codes (Quebec)** | **CCP**^b^ **(Alberta)** |  |
| --- | --- | --- | --- |
| Colonoscopy | 697, 700, 703, 863 | 01.22, 01.22A, 01.22B, 01.22C, 58.99C |  |
| Polypectomy | 749 | 57.21A |  |
| Sigmoidoscopy | 635 | 01.24A, 01.24B, 01.24BA, 0.124BB, 58.99D |  |
| Double contrast barium enema | 8160 | X 86, X 87, X 88, X 88A |  |
| **Risk factors in past 5 years** | **ICD-9**^c^ **(Quebec, Alberta)** |  |  |
| Inflammatory bowel disease | 555.x, 556.x |  |  |
| Colorectal polyps | 569.0, 211.3, 211.4 |  |  |
| Colorectal cancer | 153.x, 154.x |  |  |
| **Symptoms in the past year** | **ICD-9 (Quebec, Alberta)** |  |  |
| Rectal bleeding | 569.3, 578.1 |  |  |
| Diarrhea | 009.x, 564.5 |  |  |
| Vomiting | 787.0 |  |  |
| Weight loss | 783.1, 783.2 |  |  |
| Anemia | 280.0, 280.9, 281.0, 281.9, 285.0, 285.1, 285.8, 285.9 |  |  |
|  |  |  |  |
| **Hospitalizations in the past 5 years** | **ICD-9 (Quebec, Alberta)** | **ICD-10**^d^ **(Quebec, Alberta)** |  |
| Large bowel diseases | 555.x, 556.x, 557.x, 562.1 | K50.x, K51.x, K55.x, K57.2, K57.3, K57.8, K57.9 |  |
| **Surgeries in the past 5 years** | **CCI** ^e^ **(Quebec, Alberta)** | **CCP (Quebec)** | **ICD-9 (Alberta)** |
| Large bowel surgeries | 1NK82EN, 1NK82RR, 1NK82DN, 1NK82RE, 1NK87DN, 1NK87RE, 1NK87DX, 1NK87TF, 1NK87DY, 1NK87TG, 1NM87DF, 1NM87RN, 1NM87DE, 1NM87DN, 1NM87RD, 1NM87RE, 1NM87TF, 1NM87DX, 1NM87DY, 1NM87TG, 1NM89DF, 1NM89RN, 1NM89DX, 1NM89TF, 1NM91DF, 1NM91DE, 1NM91DN, 1NM91DX, 1NM91DY, 1NM91RN, 1NM91RD, 1NM91RE, 1NM91TF, 1NM91TG, 1NQ87RD, 1NQ87DF, 1NQ87PB, 1NQ87TF, 1NQ87DX, 1NQ87TF, 1NQ87DX, 1NQ87PN, 1NQ89SF, 1NQ89KZ, 1NQ89GV, 1NQ89RS, 1NQ89LH, 1NQ89AB, 1NQ89SFXXG, 1NQ89KZXXG, 1NQ89RSXXG, 1NQ89LHXXG | 57.41, 57.53, 57.54, 57.55, 57.56, 57.59, 57.69, 57.84, 57.85, 58.92, 58.04, 60.31, 60.39, 60.51, 60.52, 58.21, 58.22, 58.23, 58.24, 57.21, 57.29, 60.49, 58.11, 58.12, 58.13, 58.14, 58.41, 58.42, 58.43, 58.44, 58.51, 58.52, 58.53 | 48.50, 48.51, 48.52, 48.59, 45.41, 45.74, 45.71, 45.81, 45.42, 45.70, 45.71, 45.72, 45.73, 45.82, 45.43, 45.83, 45.49, 48.40, 48.41, 48.42, 48.43, 48.49, 48.60, 48.61, 48.62, 48.63, 48.64, 48.65, 48.69, 46.20, 46.21, 46.22, 46.23, 46.10, 46.11, 46.13 |

^a^ Régie de l’assurance maladie du Québec

^b^ Canadian Classification of Diagnostic, Therapeutic, and Surgical Procedures

^c^ The International Classification of Diseases, 9th Revision, Clinical Modifications

^d^ International Statistical Classification of Diseases and Related Health Problems, 10th Revision

^e^ Canadian Classification of Health Interventions
